# SIAH2-mediated and organ-specific restriction of HO-1 expression by a dual mechanism

**DOI:** 10.1038/s41598-020-59005-3

**Published:** 2020-02-10

**Authors:** Shashipavan Chillappagari, Ratnal Belapurkar, Andreas Möller, Nicole Molenda, Michael Kracht, Susanne Rohrbach, M. Lienhard Schmitz

**Affiliations:** 1 Institute of Biochemistry, Justus-Liebig-University, Member of the German Center for Lung Research, D-35392 Giessen, Germany; 2 Tumour Microenvironment Laboratory, QIMR Berghofer Medical Research Institute, Herston, QLD 4006 Australia; 3 Department of Physiology, Justus-Liebig-University, D-35392 Giessen, Germany; 4 Rudolf-Buchheim-Institute of Pharmacology, Justus-Liebig-University, Member of the German Center for Lung Research, D-35392 Giessen, Germany

**Keywords:** Biocatalysis, Chemical modification, Proteolysis

## Abstract

The intracellular levels of the cytoprotective enzyme heme oxygenase-1 (HO-1) are tightly controlled. Here, we reveal a novel mechanism preventing the exaggerated expression of HO-1. The analysis of mice with a knock-out in the ubiquitin E3 ligase seven in absentia homolog 2 (SIAH2) showed elevated HO-1 protein levels in specific organs such as heart, kidney and skeletal muscle. Increased HO-1 protein amounts were also seen in human cells deleted for the *SIAH2* gene. The higher HO-1 levels are not only due to an increased protein stability but also to elevated expression of the HO-1 encoding *HMOX1* gene, which depends on the transcription factor nuclear factor E2-related factor 2 (NRF2), a known SIAH2 target. Dependent on its RING (really interesting new gene) domain, expression of SIAH2 mediates proteasome-dependent degradation of its interaction partner HO-1. Additionally *SIAH2*-deficient cells are also characterized by reduced expression levels of glutathione peroxidase 4 (GPX4), rendering the knock-out cells more sensitive to ferroptosis.

## Introduction

Ubiquitin E3 ligases regulate the activity and turnover of many target proteins, thus controlling key features such as metabolism, stress signaling and cell cycle progression^1^. The RING family of ubiquitin E3 ligases comprises the SIAH family. The human genome encodes SIAH1 and the homologous SIAH2 protein as well as SIAH3, which lacks a functional RING domain and is only expressed in a limited subset of cancer cell lines^2^. SIAH1 and SIAH2 proteins have a largely divergent N-terminal part, but are highly conserved in the RING domain. Both E3 ligases have overlapping and distinct substrate binding abilities^3^. The enzymatic activity of SIAH proteins typically leads to poly-ubiquitination and subsequent proteasomal degradation of its client proteins, but also SIAH-mediated mono- and di-ubiquitination has been described^4–6^. SIAH function can be regulated at several levels, as these E3 ligases can form homo- and heterodimers, and also associate with regulatory proteins^4,7^. The substrate binding ability of SIAH proteins is additionally regulated by phosphorylation, which can be mediated by several kinases^8,9^. The intracellular levels of SIAH proteins are typically very low, due to ongoing auto-ubiquitination and degradation, as well as by association with ubiquitin carboxyl-terminal hydrolase 13 (USP13)^10^. The SIAH2 protein has been implicated in the regulation of many different biological processes including the control of cell metabolism by degradation of α-ketoglutarate dehydrogenase, thus shifting glutamine metabolism from oxidation to reductive carboxylation under hypoxic conditions^11^. SIAH2 contributes to control several hypoxia-regulated pathways exerted by the p53 family member p73, Hippo signaling and homeodomain-interacting protein kinase 2 (HIPK2)^8,12,13^. Several studies have shown that SIAH2 indirectly controls the abundance of the hypoxia-induced transcription factor HIF-1α by SIAH2-mediated degradation of hypoxia regulators, such as prolyl hydroxylase (PHD)1 and PHD3^14^. SIAH2 is also a relevant regulator of reactive oxygen species (ROS) metabolism, as this E3 ligase does not only regulate the cell response to low oxygen and the stability of several metabolic enzymes, but also of transcription factors such as NRF1 and NRF2, which contribute to the expression of antioxidant proteins^15,16^.

One NRF2-regulated anti-oxidative protein is heme oxygenase 1 (HO-1), an inducible and rate-limiting enzyme in the catabolism of heme. Pro-oxidative conditions, inflammation and further adverse conditions lead to increased HO-1 expression to allow HO-1-mediated removal of the pro-oxidant heme^17^. HO-1 catalyses the oxidative cleavage of heme to biliverdin, ferrous iron (Fe^2+^), and carbon monoxide^18^. This enzymatic activity is important to mediate the cytoprotective effect of HO-1 against oxidative injury. While HO-1 mostly acts as a pro-survival factor, it can also promote ferroptosis, an iron- and lipid peroxidation-dependent form of cell death^19,20^. HO-1 is found as a membrane protein in the smooth endoplasmic reticulum (sER) with most of the protein oriented towards the cytosol. Adverse conditions lead to the C-terminal cleavage of HO-1, allowing its release from the sER and the diffusion to various intracellular localizations, including the nucleus and mitochondria^21,22^.

Here, we reveal a novel role of SIAH2 as controller of oxygen homeostasis by the identification of HO-1 as SIAH2 target. The stabilization of HO-1 and the downregulation of GPX4 make *SIAH2*-deficient cells more vulnerable to ferroptosis.

## Results

### SIAH2 regulates HO-1 protein levels

**Figure 1:**
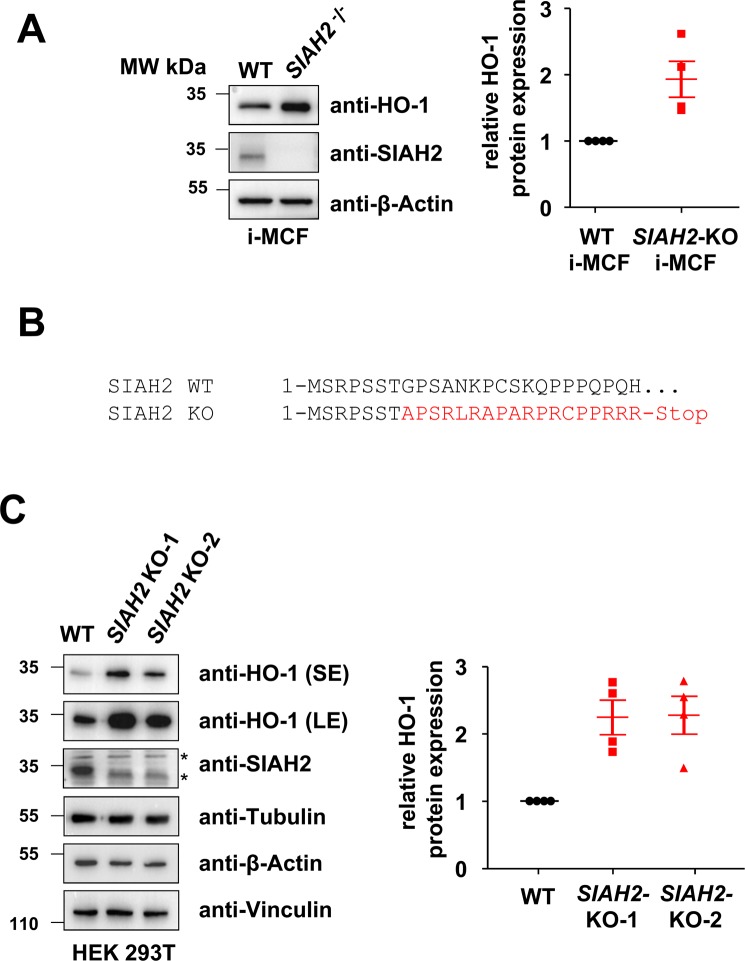
Increased levels of HO-1 protein in *SIAH2*-deficient cells. **(A)** i-MCFs from wild-type (WT) and *SIAH2* knock-out mice were lysed and equal amounts of protein contained in cell lysates was tested by Western blotting for the expression of the indicated proteins using specific antibodies. The positions of molecular weight (MW) markers are indicated. **(B)** The genomic DNA of two independent 293T cell clones engineered by CRISPR-Cas9 to contain an Indel mutation in the first SIAH2-encoding exon and defect on SIAH2 protein expression were isolated. PCR amplification of the relevant genomic region and sequencing showed the same mutation in both cell clones designated *SIAH2*-KO-1 and *SIAH2*-KO-2. The consequences of this mutation on protein translation are shown, the region undergoing a frame shift is indicated by red. **(C)** Equal numbers of 293T wild-type cells and two independently obtained derivatives thereof with a mutation in the *SIAH2* gene were lysed and tested for the expression of the indicated proteins by immunoblotting (left). The positions of non-specific bands are indicated by asterisks. The right part shows a quantification of HO-1 expression from four independent experiments. Protein amounts of HO-1 and β-Actin were quantified using the ChemiDoc Imaging System. Relative protein amounts were normalized to β-Actin and HO-1 expression in wild-type cells was set as one, the median and 25–75% quantiles are indicated. SE: short exposure, LE: long exposure.

**Figure 2:**
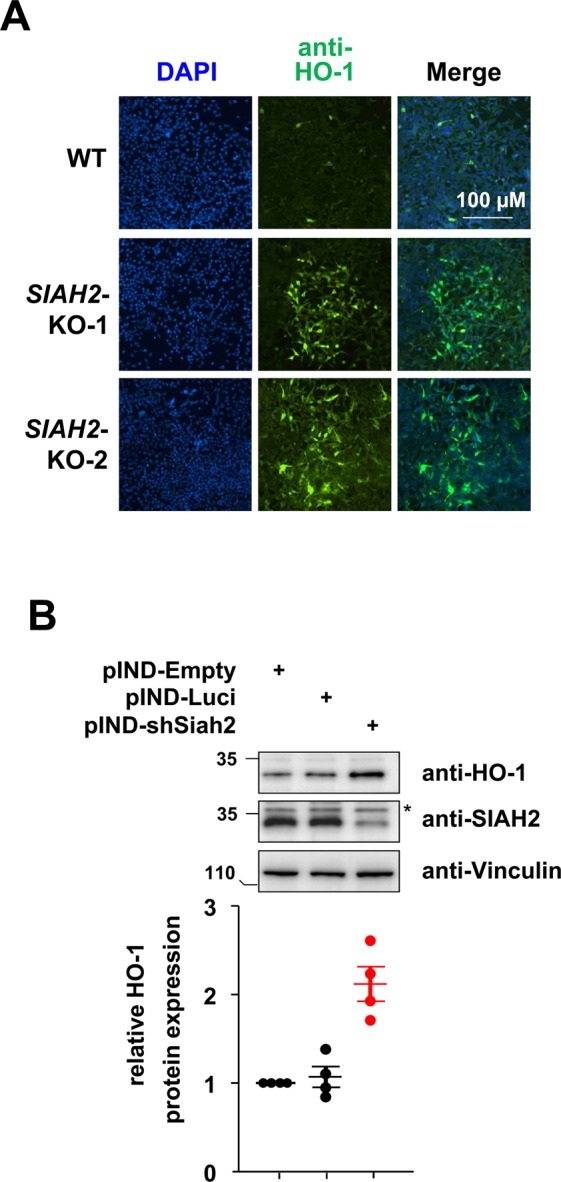
Inverse correlation of HO-1 and SIAH2 abundance. **(A)** The indicated 293T wild-type cells and two *SIAH2*-deleted cell clones were stained for HO-1 expression by indirect immunofluorescence, the nuclear DNA was stained with DAPI. **(B)** Cell clones with a stably integrated pINDUCER plasmid allowing doxycycline-inducible downregulation of luciferase (control) or SIAH2 were treated for 4 days with Dox (1 μg/ml) and analysed for expression of HO-1 and SIAH2 by Western blotting, the asterisk indicates a non-specific band. The lower part shows a quantification of four independent experiments.

The contribution of SIAH2 and also HO-1 in oxygen-dependent signalling raised the question of a mutual cross-regulation between both proteins. As a starting point, we tested HO-1 protein levels in immortalized mouse cardiac fibroblasts (i-MCF) derived from control or *SIAH2*-knock-out animals. Western blot analysis showed increased HO-1 protein levels in i-MCF cells derived from *SIAH2* knock-out mice (Fig. 1A). As the mouse and human SIAH-encoding genes show differences in sequence and gene organization^23,24^, we investigated whether this type of cross-regulation between SIAH2 and HO-1 also occurs in human cells. We used the clustered regularly interspaced short palindromic repeats (CRISPR)-Cas9 system to interfere with SIAH2 expression in human embryonic kidney (HEK)293T cells, thus generating two independent cell clones harbouring the same Indel mutant resulting in expression of only the first 7 amino acids of human SIAH2 (Fig. 1B). Comparison of these cells with wild-type controls showed an inverse correlation of HO-1 and SIAH2 protein abundance (Figs. 1C and 2A). This increase of HO-1 protein was also seen in cells expressing inducible SIAH2-specific shRNA (Fig. 2B). Collectively, these data show that reduction of SIAH2 expression results in increased HO-1 protein levels, irrespective of the method of SIAH2 downregulation or species.

### SIAH2-mediated restriction of HO-1 abundance is organ-specific

**Figure 3:**
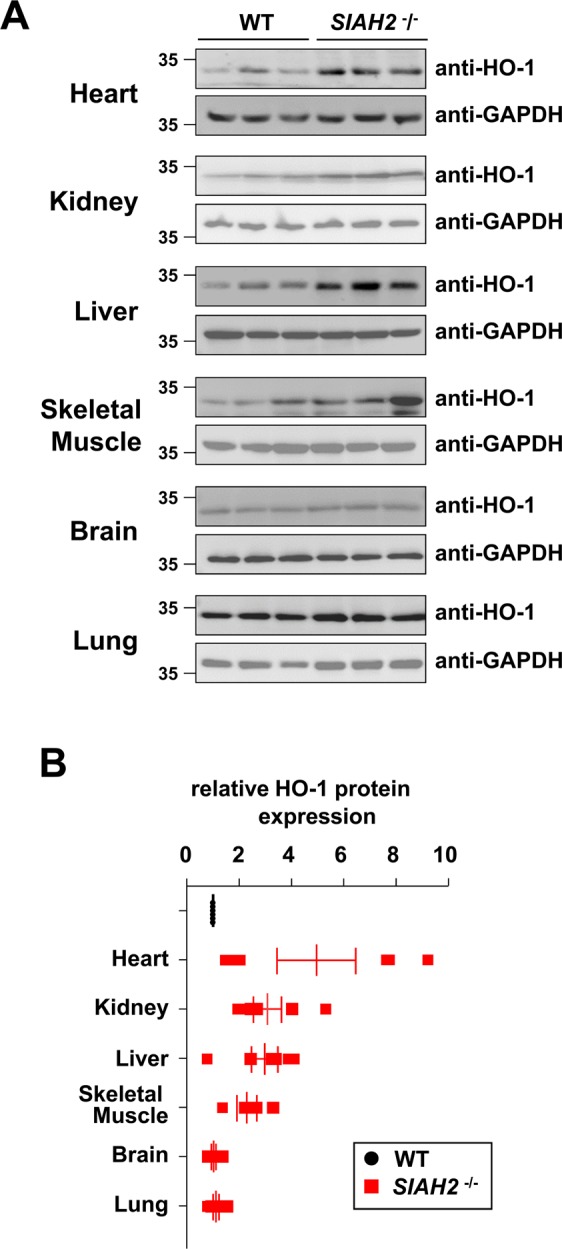
Organ-specific consequences of *SIAH2* knock-out on HO-1 abundance. Control wild-type mice or *SIAH2*^−/−^ mice were sacrificed and tissue extracts were prepared from the indicated different organs. Western blot analysis was performed to reveal relative expression of HO-1. The results in **(A)** show immunoblots from samples tested in triplicates on the same gel. **(B)** This quantification shows relative HO-1 expression from 6 different samples per genotype, relative protein amounts were normalized to GAPDH. HO-1 expression in the respective wild-type tissues was set as one, the median and 25–75% quantiles are indicated.

Next, we determined whether SIAH2-mediated control of HO-1 protein abundance occurs in all organs. Heart, kidney and liver samples from *SIAH2* knock-out mice presented with elevated HO-1 abundance compared to wild-type organs (Fig. 3A), consistent with the results we obtained from murine i-MCFs. A quantitative analysis of Western blot signals from multiple experiments showed only slightly increased HO-1 levels in skeletal muscle, but unchanged HO-1 abundance in the lung and brain of *SIAH2*-deficient animals (Fig. 3B). Collectively, these data suggest that the suppressive effects of SIAH2 on HO-1 protein abundance are confined to specific organs and probably also cell types.

### SIAH2 loss results in increased HO-1 mRNA and protein abundance

**Figure 4:**
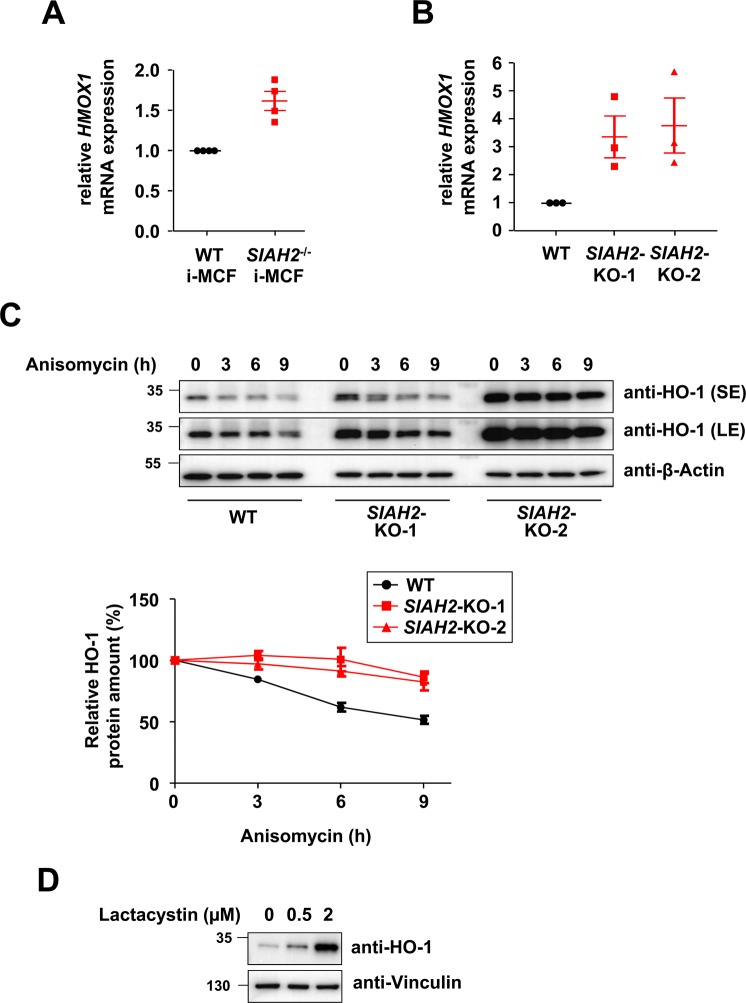
Increased levels of HO-1 mRNA and elevated HO-1 protein stability in *SIAH2*-deficient cells. **(A)** i-MCFs from wild-type and *SIAH2* knock-out mice were analysed for expression levels of *HMOX1* mRNAs by RT-qPCR, results from 4 experiments are shown. **(B)** The experiment was done as in (**A**) with the difference that *HMOX1* mRNAs from wild-type and *SIAH2* knock-out 293T cells were used. **(C)** The indicated cell lines were treated with Anisomycin (5 µM) for different periods as shown and protein expression of HO-1 was analysed by immunoblotting, a long exposure (LE) and short exposure (SE) is displayed. The lower part shows HO-1 decay curves from three experiments, standard deviations are shown. **(D)** 293T cells were treated for 8 h with lactacystin and HO-1 expression was determined by immunoblotting.

To test whether the increased HO-1 protein levels correlate with increased mRNA levels, we performed RT-qPCR experiments. We found elevated HO-1 encoding mRNAs in *SIAH2*-deficient i-MCFs and 293T cells (Fig. 4A,B), suggesting an impact of SIAH2 on the expression of the *HMOX1* gene. In order to test a potential influence of SIAH2 on HO-1 protein stability, *de novo* protein synthesis was blocked by Anisomycin and HO-1 decay monitored over 9 h. The quantitative analysis of these experiments detected increased protein stability of HO-1 in *SIAH2*-deficient cells (Fig. 4C). As SIAH2 can act as an ubiquitin E3 ligase, we tested whether HO-1 levels are controlled by the ubiquitin/proteasome system. Inhibition of the proteasome by lactacystin dose-dependently resulted in increased HO-1 protein levels (Fig. 4D), showing the importance of constitutive HO-1 degradation by the ubiquitin/proteasome system.

### SIAH2 targets HO-1 for degradation

**Figure 5:**
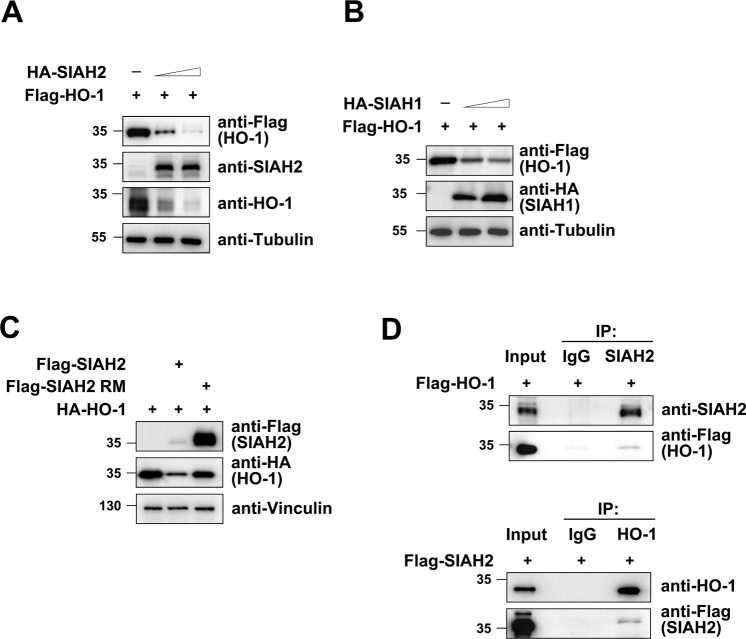
SIAH2-mediated downregulation of its interaction partner HO-1. **(A)** 293T cells were transfected to express increasing amounts of HA-SIAH2 along with Flag-tagged HO-1 as shown. Two days later cells were harvested and analysed by Western blotting as shown. **(B)** The experiment was done as in (**A**) with the exception that increasing amounts of HA-SIAH1 were expressed. **(C)** 293T cells were transfected to express HA-tagged HO-1, either alone or together with wild-type SIAH2 or a SIAH2 RING domain mutant (RM). The relative expression of proteins is shown, please note that the SIAH2 RM shows an increased expression level due to the lack of auto-ubiquitination and degradation. **(D)** Lysates from 293T cells were used for immunoprecipitation (IP) using antibodies for SIAH2 (upper) or HO-1 (lower) in combination with control IgGs. The precipitated samples were analysed by Western Blotting using appropriate antibodies, input samples ensured correct protein expression.

To test whether SIAH2 expression leads to HO-1 degradation, 293T cells were transfected to express Flag-tagged HO-1 alongside increasing amounts of HA-SIAH2. We found a dose-dependent reduction of HO-1 protein levels inversely correlated with increasing amounts of SIAH2 (Fig. 5A). SIAH1 and SIAH2 have overlapping but also distinct substrates. To test if SIAH1 is also capable of controlling HO-1 abundance, we expressed increasing SIAH1 alongside HO-1. Similar to SIAH2, SIAH1 is able in a dose-dependent fashion to reduce HO-1 protein levels (Fig. 5B), although HO-1 degradation was less efficient and complete. SIAH2-mediated target degradation commonly depends on an intact RING domain, which binds E2 proteins and is important for auto- and trans-ubiquitination^25^. Expression of the wild-type, but not of a RING-mutated version of SIAH2 (SIAH2 RM), resulted in the reduced abundance of HO-1 (Fig. 5C), albeit the SIAH2 RM being expressed at much higher levels due to defective auto-ubiquitination. These data suggest that SIAH2 causes, dependent on the functionality of its RING domain, reduced HO-1 levels, raising the possibility that it serves as a direct ubiquitin E3 ligase for HO-1. To test for a possible interaction between SIAH2 and HO-1, co-immunoprecipitation experiments were performed. Immunoprecipitation of the endogenous SIAH2 protein allowed the specific detection of co-immunoprecipitated HO-1, while *vice versa* SIAH2 was found in association with the immunoprecipitated endogenous HO-1 protein (Fig. 5D). Together, this data shows that SIAH2 and HO-1 interact, and this interaction results in the proteasomal degradation of the HO-1 protein.

### Functional consequences of SIAH2-mediated HO-1 degradation

**Figure 6:**
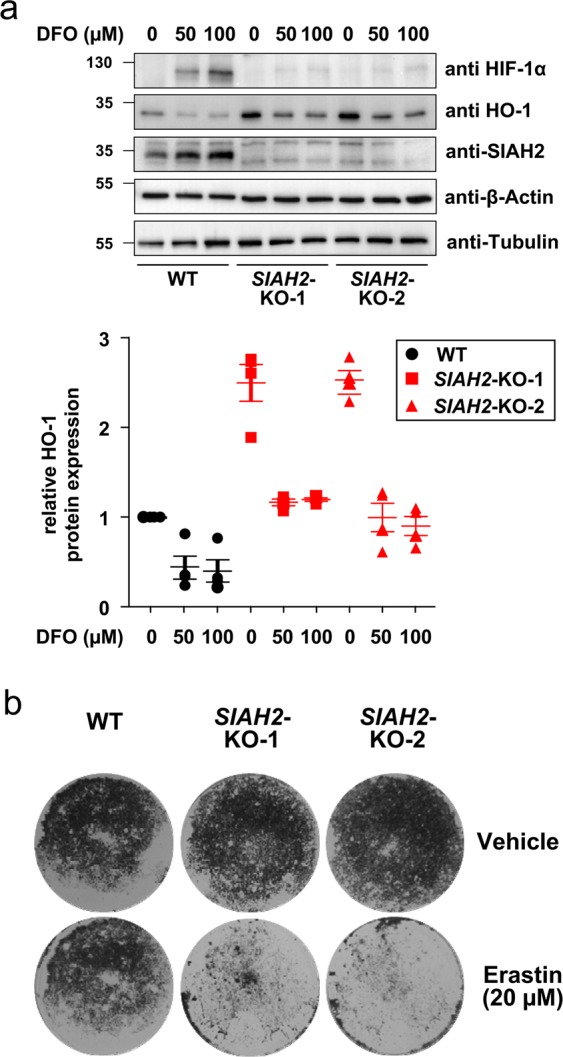
Functional analysis of SIAH2-mediated HO-1 degradation. **(a)** 293T wild-type and *SIAH2* knock-out cells were treated with the indicated DFO concentrations for 8 h, followed by analysis of HIF-1α, SIAH2 and HO-1 expression by Western blotting. The lower part shows a quantification of HO-1 expression from three independent experiments. **(b)** The indicated cell lines were treated with DMSO as a control or with erastin to induce ferroptosis. After two days, the surviving cells were grown to colonies and stained with crystal violet, one out of three independent experiments with similar results is shown.

To elucidate the physiological and functional consequences of the SIAH2/HO-1 interaction, we first investigated the hypoxic response pathway as both proteins have been shown to be involved. 293T wild-type and *SIAH2*-deficient cells were treated with the iron chelator deferoxamine (DFO), a chemical hypoxia-mimetic. DFO-dependent stabilization of HIF-1α was only seen in wild-type cells, but not in *SIAH2*-deficient cells (Fig. 6a), in line with previous publications^26^. Additionally, consistent with the literature we also observed a reduced HO-1 expression in DFO-treated cells^27^, which also occurred in *SIAH2*-deficient cells (Fig. 6a). These results suggest that SIAH2 is unlikely to participate in DFO-dependent HO-1 degradation.

Another HO-1 regulated process is ferroptosis, a regulated but non-apoptotic form of cell death that can be induced experimentally by compounds such as erastin^28^. Ferroptosis is characterized by high ROS levels derived from iron metabolism and lipid peroxidation. Depending on the stimulus and cell type, HO-1 can mitigate or enhance ferroptosis^19,29,30^. To test the effects of *SIAH2* deletion on erastin-induced ferroptosis, wild-type and *SIAH2*-deficient cells were used. Two days after induction of ferroptosis, dead cells were aspirated off and the surviving cells were grown to colonies. Trypan blue staining of the surviving cell clones showed that *SIAH2*-deficient cells were more susceptible to ferroptosis (Fig. 6B), raising the possibility that elevated HO-1 levels contribute to enhanced ferroptosis in the knock-out cells. Similar results were obtained after induction of ferroptosis with RSL3, an agent that induces ferroptosis via inhibition of GPX4 (Suppl. Fig. S1)^31^. The occurrence of ferroptosis in response to erastin and RSL3 treatment was confirmed by the cell protective function of Ferrostatin (Suppl. Fig. S2), a known suppressor of this form of cell death^32^.

**Figure 7:**
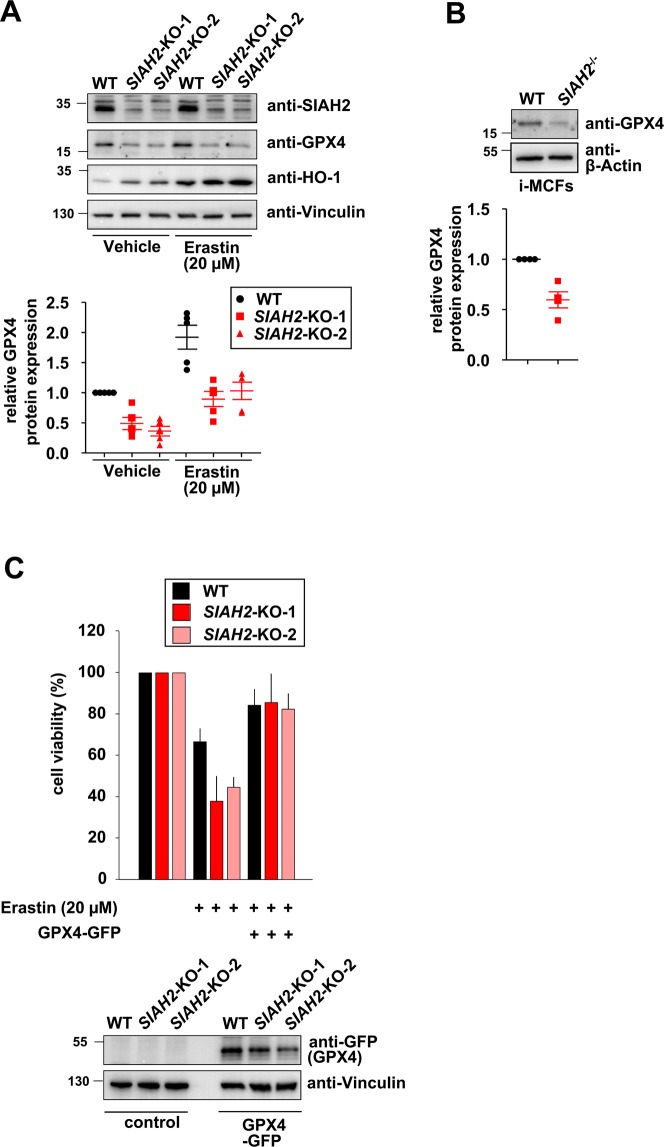
Effect of SIAH2-regulated GPX4 expression on ferroptosis. **(A)** The indicated cells were treated with DMSO or erastin and two days later cell extracts were prepared to measure expression of the indicated proteins by immunoblotting. The lower part shows a quantification of GPX4 expression. **(B)** The experiment was done as in (A) with the exception that GPX4 expression was determined in untreated wildtype and *SIAH2* knock-out i-MCFs. **(C)** The indicated cells were transfected to express GFP-GPX4. The next day, a fraction of the cells was lysed and used for immunoblotting to determine GFP-GPX4 expression (lower). The other cells were treated with erastin for 40 h, followed by determination of cell death using the PrestoBluecell viability reagent (upper). Error bars show SEM from two experiments measured in triplicates.

To monitor the differential sensitivity to ferroptosis by an alternative experimental approach, we determined the levels of glutathione peroxidase 4 (GPX4), which inhibits ferroptosis and is a widely used marker for this process^31^. Increased sensitivity of SIAH-2-deficient cells to ferroptosis was mirrored at the level of diminished GPX4 protein expression in untreated, erastin-treated (Fig. 7A) and RSL3-treated *SIAH2*-deficient cells (Suppl. Fig. S3). This reduced expression was not reflected at the mRNA level (Suppl. Fig. S4), suggesting SIAH2-dependent GPX4 regulation at the posttranscriptional level. Differential GPX4 expression also occurred in wild-type and *SIAH2* knock-out i-MCFs (Fig. 7B), showing that SIAH2-dependent expression changes of GPX4 is reproducible across species and cell lines. Re-expression of GPX4 largely rescued *SIAH2*-deficient cells from increased ferroptosis (Fig. 7C), suggesting a contribution of GPX4 for the ferroptosis-antagonizing function of SIAH2.

## Discussion

Here, we show that elimination or downregulation of SIAH2 expression by genetic knock-out, CRISPR-Cas9-mediated inactivation or shRNA-mediated knockdown leads to elevated HO-1 protein levels. This increase is also reflected at the mRNA level and readily explained by increased stability of transcription factor NRF2, a known SIAH2 target and master regulator of HO-1 expression^16,33^. It will be interesting to study whether SIAH2 only contributes to the control of basal HO-1 turnover in unstressed cells, or whether it also participates in signal-regulated HO-1 degradation pathways.

Although HO-1 *per se* is mainly cytoprotective, permanently elevated levels have negative physiological effects, as revealed by the analysis of transgenic mice overexpressing this enzyme. Lung-specific overexpression of HO-1 causes lower amounts of LPS-induced cytokine levels, thus impairing the immune response^34^. In addition, HO-1 overexpression causes neurological disorders and enhances carcinogenesis^35–37^. Also lack of HO-1 expression is detrimental, as mice with a global HO-1 knock-out show a reduced stress defense and iron reutilization^38,39^, showing the necessity to appropriately balance HO-1 expression and protein abundance. This is achieved by multiple layers of regulation, including control of *de novo* transcription, but also through regulation of HO-1 mRNA deadenylation and turnover^40^. HO-1 protein is regulated at various levels, including the occurrence of posttranslational modifications, such as phosphorylation and ubiquitination^41,42^. In addition, the protein stability of HO-1 is positively regulated by the 14-3-3-ζ protein^43^ and downregulated by valproic acid, an anti-epileptic drug that inhibits histone deacetylases^44^.

This study shows that also overexpression of SIAH1 causes a reduction in HO-1 protein levels, but it remains unclear whether this effect is mediated by its known ability to associate with SIAH2^3^. The differential contribution of various distinct ubiquitin E3 ligase and the occurrence of further mechanisms controlling HO-1 protein homeostasis could potentially explain the surprising finding that *SIAH2* knock-out results in organ-specific effects on HO-1 protein levels and potentially occurs with a high volatility. This finding could also be attributable to differences in the relative amount or intracellular localization of HO-1, as for example mitochondrial HO-1 would escape from degradation of the SIAH2 protein, which locates to the cytosol and nucleus. Alternatively, these organ-specific effects could also be due to differences in relative SIAH2 protein expression. The low expression of the SIAH2 protein did not allow its mass spectrometric detection in mouse organs (https://www.ebi.ac.uk/gxa/home), while *SIAH2* and *SIAH1a/b* encoding mRNAs were present in all organs at different levels (Suppl. Fig. S5) (http://biogps.org),

The increased vulnerability of *SIAH2* knock-out cells to ferroptosis is probably due to several factors, including the increased expression of pro-ferroptotic HO-1 and the decreased expression of GPX4, a key factor for this iron-catalysed necrotic pathway. Ferroptosis is specifically suited for the elimination of cancer cells, which are more vulnerable to ferroptosis, as they typically have increased iron demand compared with normal, non-cancer cells^45^. As inhibition of GPX4 activity exhibit a high selectivity and potency in killing of specific cancers, such as clear-cell carcinomas^46^, an increased understanding of the mechanisms controlling GPX4 levels will be important.

## Materials and Methods

### Antibodies, plasmids and treatments

All the information is given in the Supplementary Table 1.

### Cell culture and transfections

293T (ATCC CRL-3216), Phoenix ECO (ATCC CRL-3214) and i-MCF cells (generated for this study by immortalisation of primary mouse cells as described below) were cultured in Dulbecco’s Modified Eagle Medium (DMEM) medium supplemented with 10% fetal calf serum (FCS), Glutamine, Penicillin and Streptomycin (P/S) and occasionally in MycoZapPlus. Cells were grown at 37 °C and 5% CO_2_ and counted prior to plating using a LUNA II automated cell counter according to manufacturer’s instructions. All the transfections were performed using linear polyethylenimine (PEI) as published^47^.

### RNA extraction and qRT-PCR analysis

RNA was extracted using RNeasy kit from Qiagen as described in the manufacturer’s instructions. Concentration of RNA was measured using Eppendorf-Biophotometer, 1 µg of RNA was used for cDNA synthesis using the PrimeScript RT Master Mix from Takara Bio Inc. Generated cDNA was diluted 1:10 using sterile RNase free water and equal volumes were used as a template for amplification in a qRT-PCR. Amplification was performed using intron-flanking primers in 2X Absolute qPCR SYBR Green ROX mix (Thermo) using a Applied Biosystems 7300 device. Every reaction was performed as duplicates and quantified with the ΔΔC_T_-method. Therefore, threshold cycles (C_T_) of target genes were normalized to a housekeeping gene (*ACTB*). The resulting ΔC_T_ were compared to control samples and relative mRNA expression was calculated by R = 2^−ΔΔC^_T_.

### Protein extraction and Western blotting

Cells were washed in PBS and lysed in appropriate volume of RIPA buffer containing 1% (v/v) NP-40, 0.1% (w/v) SDS, 50 mM Tris/HCl (pH 7.4), 150 mM NaCl, 1 mM EDTA, 0.5% (w/v) sodium deoxycholate and freshly added 1 mM phenylmethylsulfonylfluoride, 10 mM NaF, 0.5 mM leupeptin (10 µg/ml), aprotinin (10 µg/ml). After incubation for 20 min on ice, cells were sonicated twice for 20 seconds. Total protein quantification was performed using a PierceBCA protein assay kit, according to manufacturer’s instructions. The lysate was mixed with 5x SDS sample buffer and the extracts were heated for 5 min at 95 °C, followed by separation of proteins using SDS-PAGE and Western blotting as described previously^47^.

Tissues from wild-type mice on a C57BL/6J background or *SIAH2* knock-out mice^48^ were homogenized in RIPA buffer containing protease and phosphatase inhibitor. Protein lysates were sonicated, cleared, and protein concentration measured. 20 μg of protein were loaded on a SDS-PAGE gel and further processed as described above. Organ harvesting was performed according to the regional authorities and ethics committees for animal research.

### Isolation of mouse cardiac fibroblasts

Hearts were excised under deep anesthesia, transferred rapidly to ice-cold saline, and mounted on the cannula of a Langendorff perfusion system as described in greater detail previously^49^. Hearts were perfused first for 10 min in a non-re-circulating manner with a calcium-free perfusion buffer, then for 20–25 min in a re-circulating manner in a buffer supplemented with collagenase and 25 µmol/l calcium. Thereafter, ventricular tissue was minced and incubated for another 5 min in re-circulating buffer. The remaining cell solution was filtered through a 200 µm nylon mesh. The suspension was centrifuged at 25 × g for 10 min to pellet down the cardiomyocytes. The remaining cells (supernatant after centrifugation) were centrifuged at 250 × g for 10 min and the pellet was resuspended in 1 ml of endothelial cell basal medium (PromoCell) and incubated with magnetic beads (ThermoFisher) pre-coated with anti CD31 for 1 hour at 4 °C with end-to-end rotation. The microvascular endothelial cells coupled to magnetic beads were separated with a magnet, washed with endothelial cell basal medium, and seeded in 35 mm culture dishes. This procedure removed over 95% of endothelial cells from the mixture and the remaining cells were seeded as cardiac fibroblasts in M199 medium supplemented with 10% fetal calf serum (FCS). All animals were handled in accordance with the NIH Guide for the Care and Use of Laboratory Animals (NIH Publication No. 85-23, 1996).

### Immortalization of primary mouse cardiac fibroblasts

Phoenix ECO cells were transfected with mTert-pBabe-puro, a retroviral plasmid encoding mouse telomerase reverse transcriptase (mTERT). Two days after transfection, the supernatant containing mTERT-encoding Retroviruses were passed through 0.45 µM sterile filters and polybrene was added to a final concentration of 5 µg/ml. The filtrate was pre-mixed with complete medium in different ratios (1:1, 2:1 and 4:1 respectively) and added to freshly isolated primary mouse cardiac fibroblasts. Two days after viral transduction cells were washed twice with PBS and further incubated in complete DMEM medium containing puromycin (2 µg/ml).

### Immunofluorescence

Cells were washed with PBS and fixed in 4% (v/v) formaldehyde solution for 10 min, followed by 2x washing with PBS. Permeabilization of the fixed cells were performed using 0.5% (v/v) Triton X-100 for 10 min and subsequently washed twice with PBS and cells were blocked using 5% (w/v) BSA for 1 hour. After addition of the first antibody (1:500 dilution) in PBS containing 1% (w/v) BSA and incubation overnight at 4 °C, the cells were washed three times with PBS. After incubation with the anti-rabbit antibody conjugated with Alexa Fluor 488 dye (1:1000) in PBS containing 1% (w/v) BSA the cells were further incubated for 1 hr in the dark. After three more washing steps the nuclei were stained with 4′,6-diamidino-2-phenylindole (DAPI) contained in the mounting medium (VECTASHIELD Mounting Medium with DAPI). Microscopy was performed using a Nikon Eclipse TE2000-E Semi-Motorized inverted fluorescence microscope and data were processed using ImageJ software.

### Co-immunoprecipitation

Cells were washed once with PBS and lysed with Pierce-IP lysis buffer. 10% of the lysate was taken as input, while the remaining material was used for co-immunoprecipitations using tosylactivated dynabeads coupled with 1 µg of the respective antibodies. After incubation in IP buffer containing 5 µM of the proteasome inhibitor lactacystin, the supernatant was removed on a magnetic stand and the pellet was washed three times with PBS containing 0.1% (W/V) Triton X-100. Bound proteins were eluted in 1x SDS sample buffer and further used for immunoblotting.

### Generation of CRIPR-Cas9 knock-out cells

Oligonucleotides targeting the first exon of the human *SIAH2* gene were cloned into pX459. These plasmids were transfected into 293T cells, followed by selection with Puromycin (1 µg/ml) for 3 days. Surviving cells were grown to colonies, picked and further expanded. Expression of SIAH2 and Cas9 was tested by Western blotting. Cell clones showing absent SIAH2 and Cas9 protein expression were further characterized by isolation of genomic DNA using the NucleoSpin Tissue kit according to the protocol of the manufacturer (Macherey-Nagel). Following PCR amplification of the genomic region encompassing the expected mutation, the PCR product was excised from an agarose gel and one of the PCR primers was directly used for sequencing of the PCR product.

### Colony formation assays

Equal numbers of 293T wild-type and *SIAH2* knock-out cells were seeded in 6-well plates until they reached 60–70% confluency. The next day, cells were treated either with erastin (20 µM) or vehicle (DMSO) and further incubated for two days at 37 °C. After removal of dead cells and washing with PBS, surviving cells were further grown several days to colonies that were stained with crystal violet solution. Images of the dried cell culture plates were captured by placing them on Kaiser slimlite photo light device using rainbow-TV lens S6x11. Images were processed using deVision Software.

### Determination of cell viability

Cell viability was evaluated using the PrestoBluecell viability reagent (Invitrogen) according to manufacturer’s instructions. Equal cell numbers were plated in flatbottom microtiter plates. After induction of cell death, 10 µl of 10-fold ready to use PrestoBluereagent was added to each well. The plate was incubated for 1 h at 37 °C in the dark and absorbance was measured using a Tecan Infinite Pro plate reader.

### Statistical analysis

All data are expressed as median, ±SD of at least three or more independent biological replicates in different experimental set-ups. Relative amounts of proteins were determined using a ChemiDoc Imaging System using Image Lab Software. All normalizations were performed with the respective house keeping gene β-Actin, Tubulin, Vinculin or GAPDH and compared with the untreated or wild-type cells, accordingly.

## Supplementary information

Supplemental Information.

Supplemental Information2.
