# Supplementary material for: SIAH2-mediated and organ-specific restriction of HO-1 expression by a dual mechanism
Source: Sci Rep. 2020 Feb 10;10:2268. doi: 10.1038/s41598-020-59005-3 (PMC7010731; doi:10.1038/s41598-020-59005-3)
Supplement: Supplementary file 1 — Supplemental Information. [file 41598_2020_59005_MOESM1_ESM.pdf]

Supplementary Material

SIAH2-mediated and organ-specific restriction of HO-1 expression by a dual mechanism

Shashipavan Chillappagari, Ratnal Belapurkar, Andreas Möller, Nicole Molenda, Michael Kracht, Susanne Rohrbach and M. Lienhard SCHMITZ

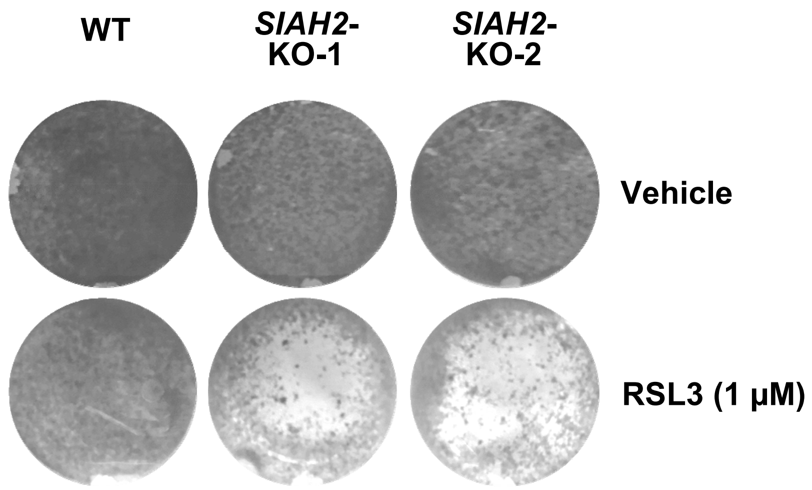

Suppl. Fig. S1. The indicated cell lines were treated with DMSO as a control or with RSL3 to induce ferroptosis. After two days, the surviving cells were grown to colonies and stained with crystal violet, one out of three independent experiments with similar results is shown.

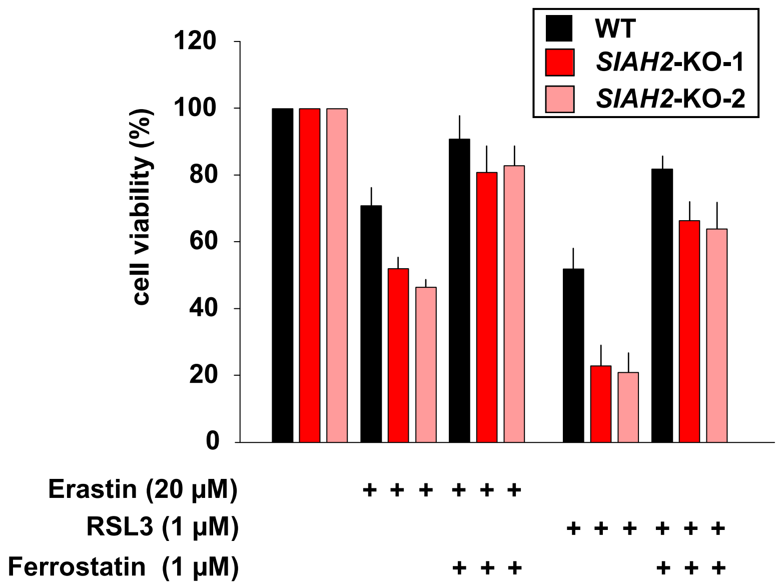

Suppl. Fig. S2. Equal numbers of 293T WT, *SIAH2*-KO-1 and *SIAH2*-KO-2 cells were plated on microtiter plates and pretreated with either ferroptosis inhibitor ferrostatin-1 or vehicle control. Two h later, erastin or RSL3 were added for 40 h and cell death was scored using the PrestoBlue cell viability reagent. Viability of untreated cells was set to 100%, error bars show SEM from two experiments measured in triplicates.

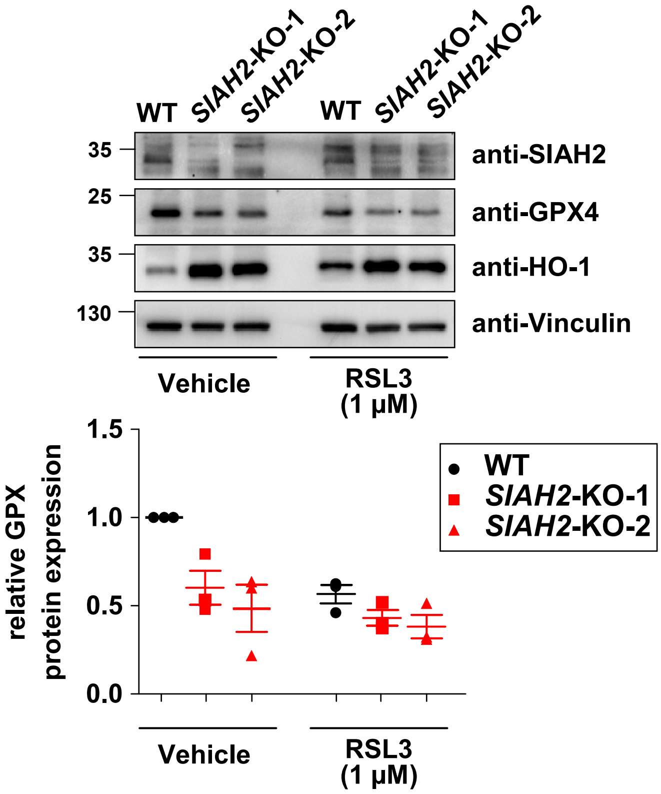

Suppl. Fig. S3. The indicated cells were treated with DMSO or RSL3 and two days later cell extracts were prepared to measure expression of the indicated proteins by immunoblotting. The lower part shows a quantification of GPX4 expression. The expression of GPX4 in untreated controls was set as 1.

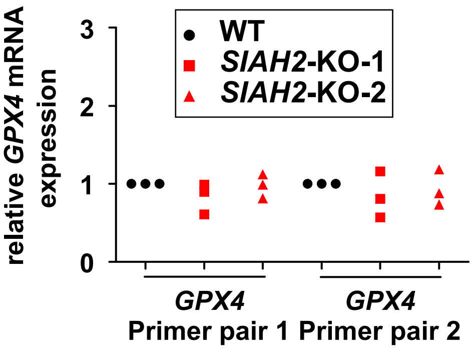

Suppl. Fig. S4. The indicated cells were analyzed for *GPX4* mRNA levels by RT-qPCR. Values were normalized to the housekeeping gene ß-Actin (*ACTB*). *GPX4* mRNA expression in control cells was arbitrarily set as 1, individual data points from the measurement in triplicates are shown.

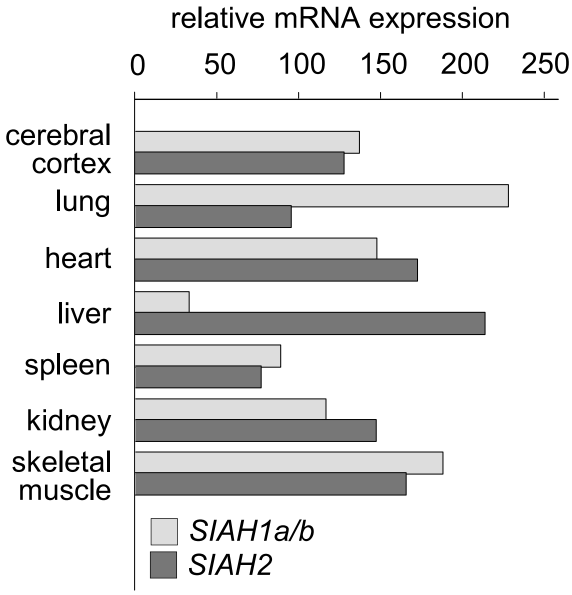

Suppl. Fig. S5. Relative *SIAH* mRNA levels in the indicated organs were retrieved from high-throughput gene expression profiling data sets (http://ds.biogps.org/?dataset=GSE10246&gene=20439).

Figure 1A

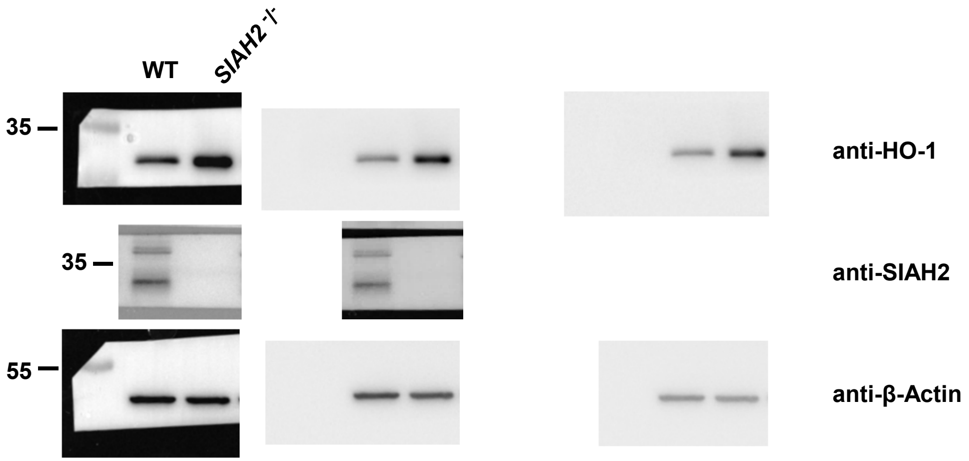

Figure 1C

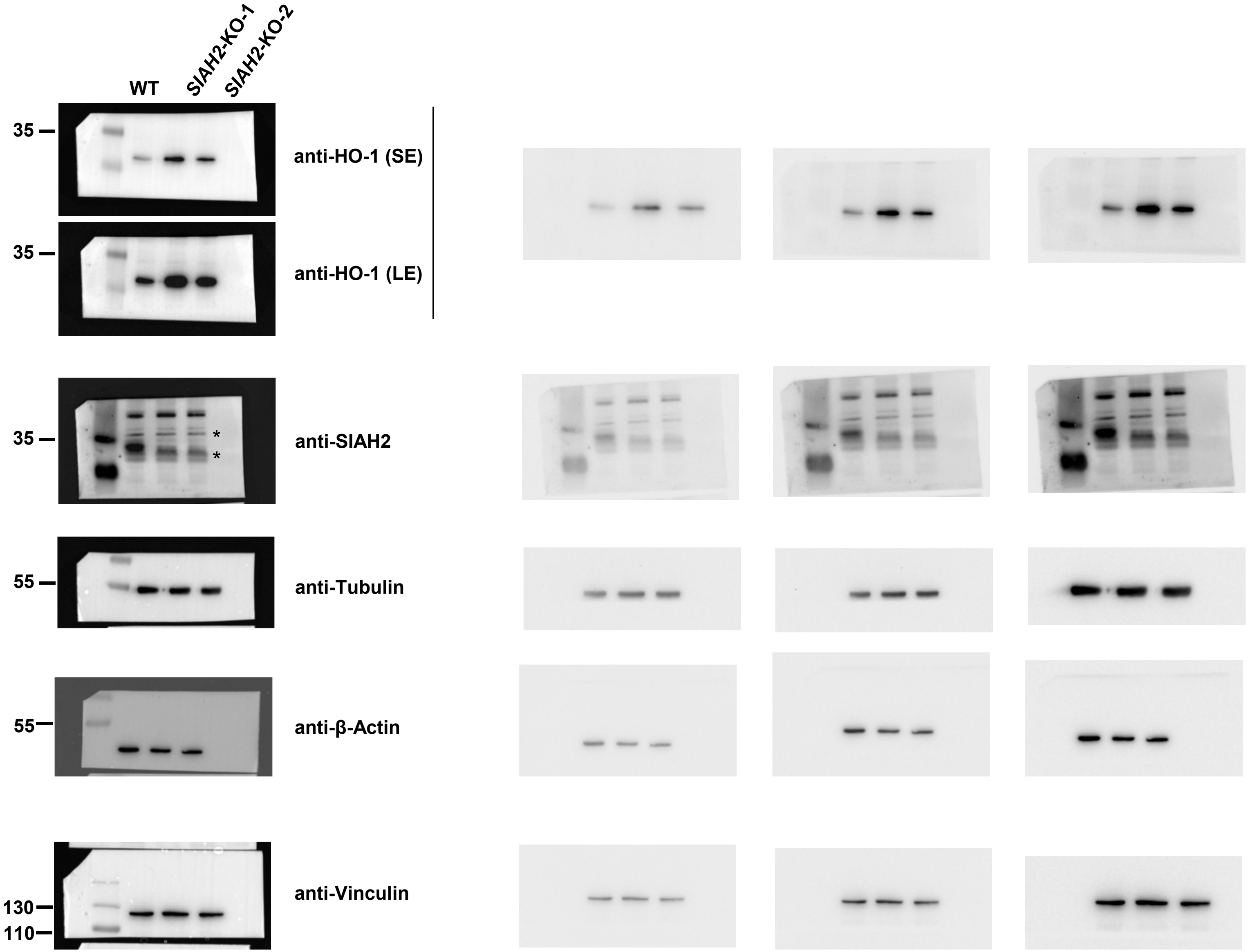
WT
SIAH2-KO-1
SIAH2-KO-2
35
anti-HO-1 (SE)
35
anti-HO-1 (LE)
35
*
*
anti-SIAH2
55
anti-Tubulin
55
anti-β-Actin
130
110
anti-Vinculin

Figure 2B

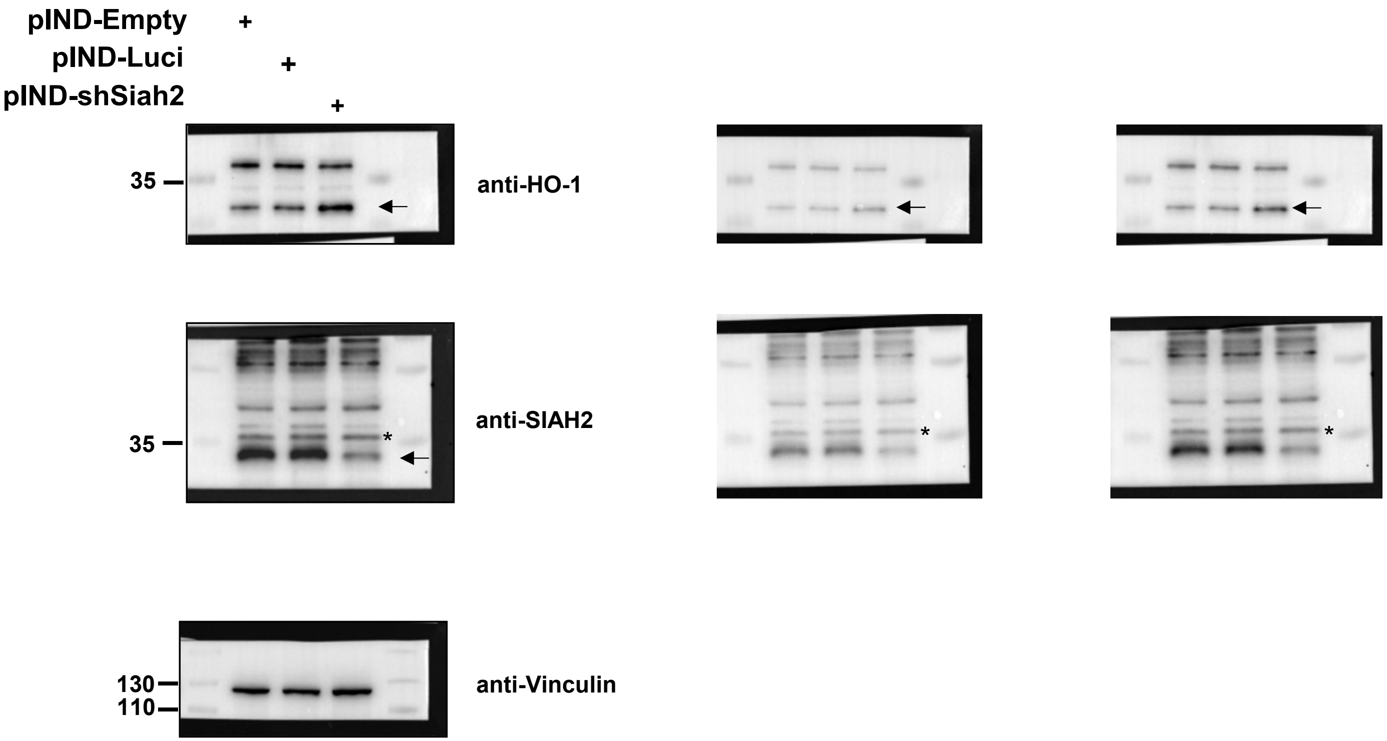

pIND-Empty +
pIND-Luci +
pIND-shSiah2 +
35
anti-HO-1
anti-SIAH2
*
130
110
anti-Vinculin

Figure 3A

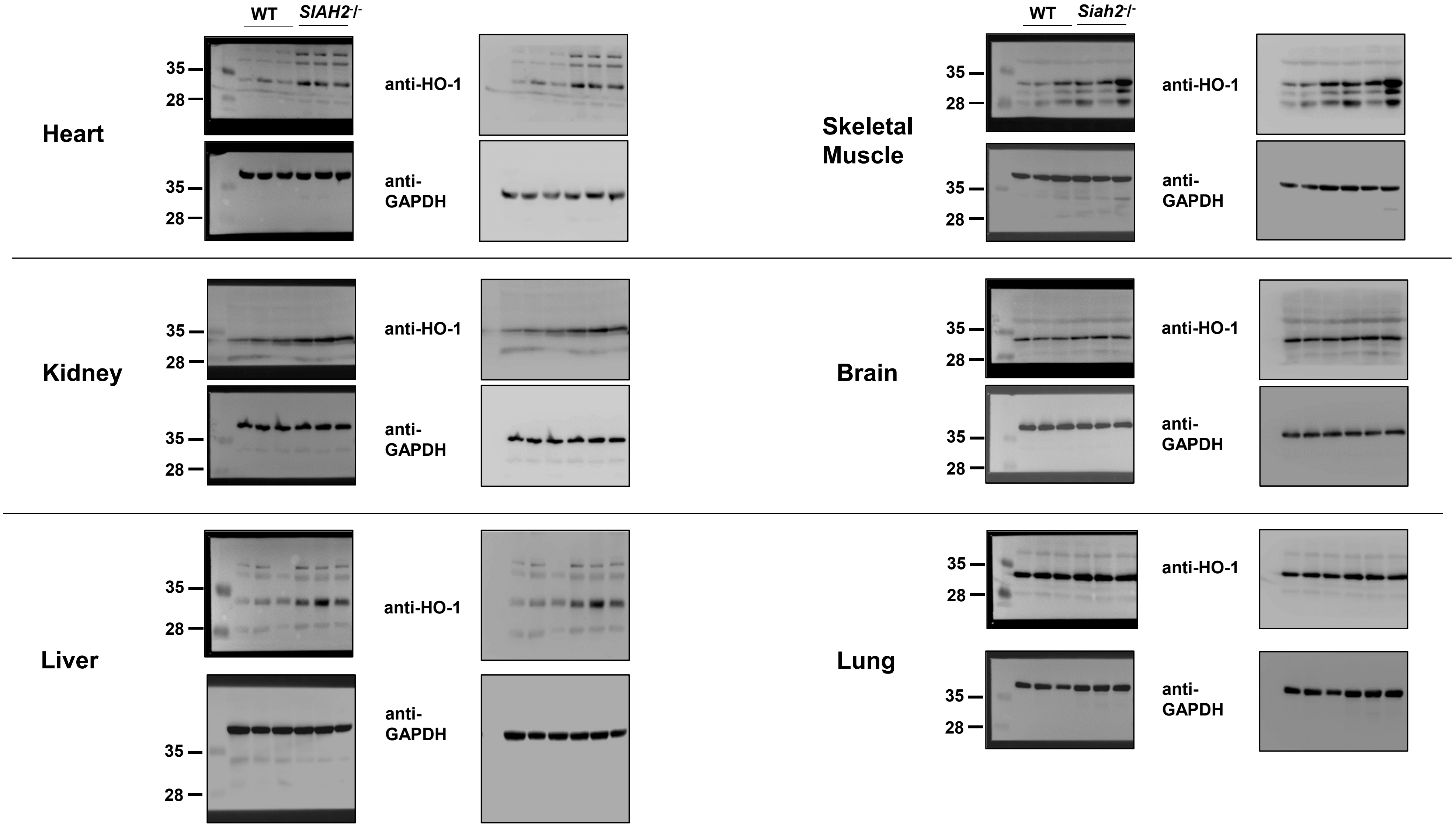
WT
SIAH2-/-
Siah2-/-
Heart
Skeletal Muscle
Kidney
Brain
Liver
Lung
anti-HO-1
anti-GAPDH
35
28

Figure 4C

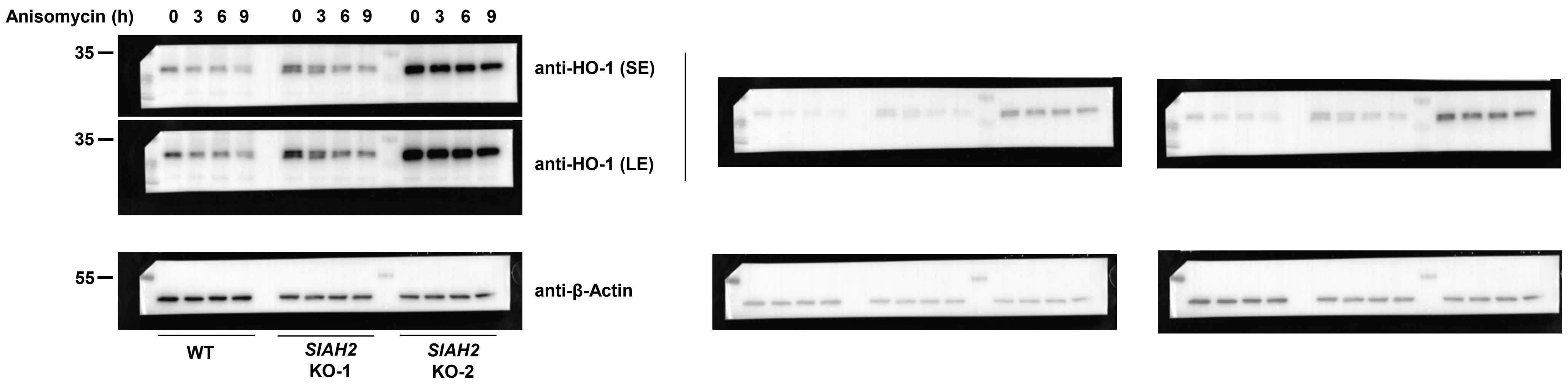

Figure 4D

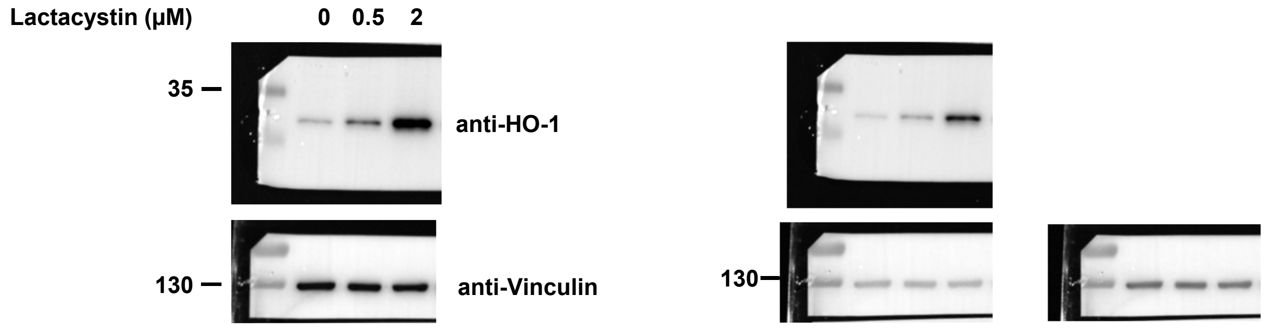

Figure 5A

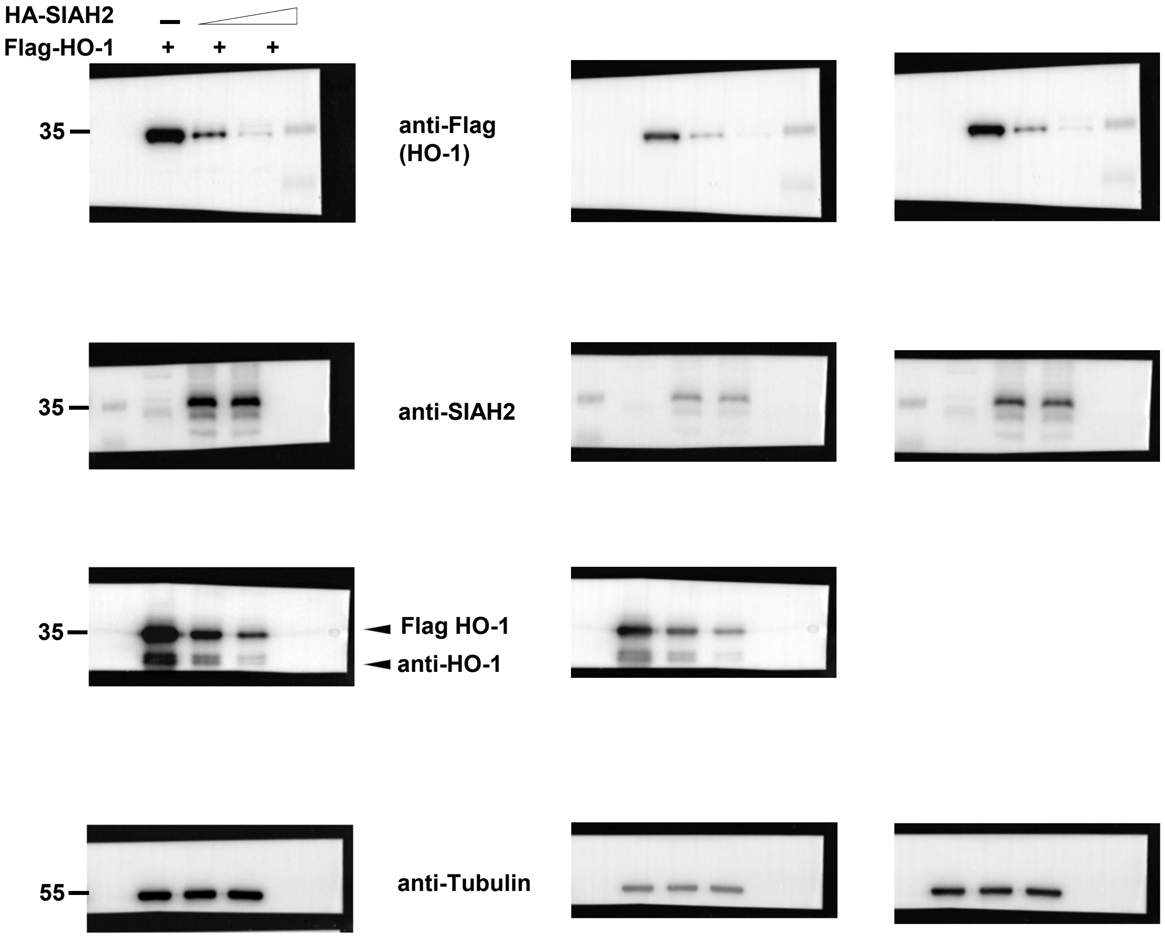
HA-SIAH2
Flag-HO-1
+
+
+
35
anti-Flag
(HO-1)
35
anti-SIAH2
35
Flag HO-1
anti-HO-1
55
anti-Tubulin

Figure 5B

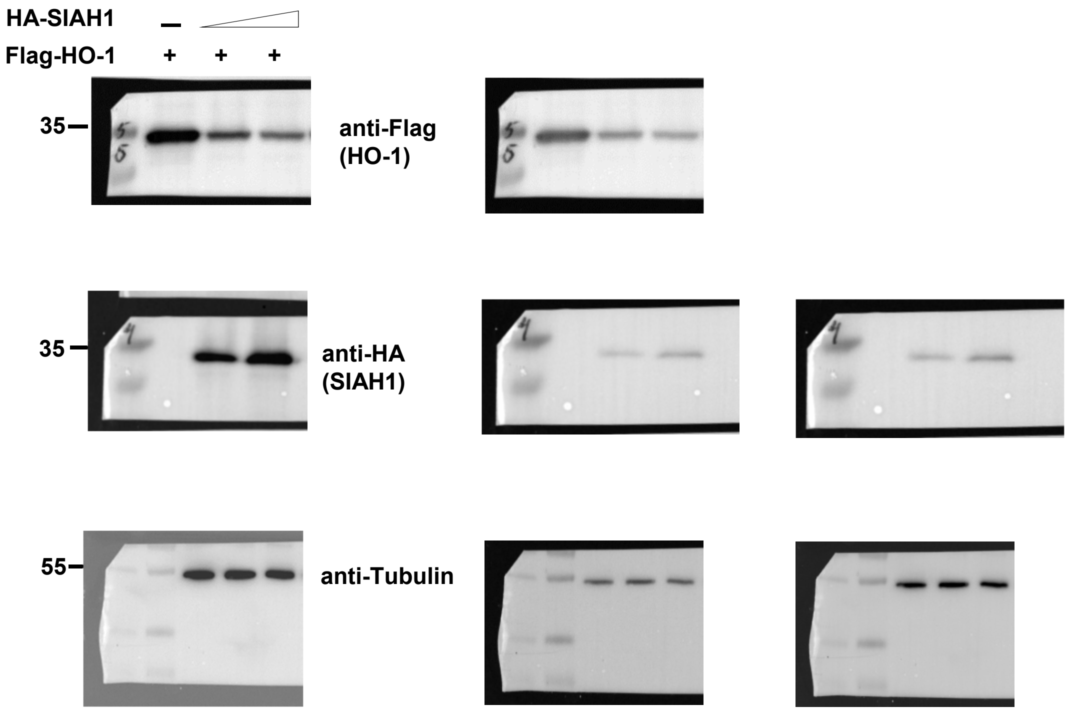
HA-SIAH1
Flag-HO-1
+ + +
35
anti-Flag
(HO-1)
35
anti-HA
(SIAH1)
55
anti-Tubulin

Figure 5C

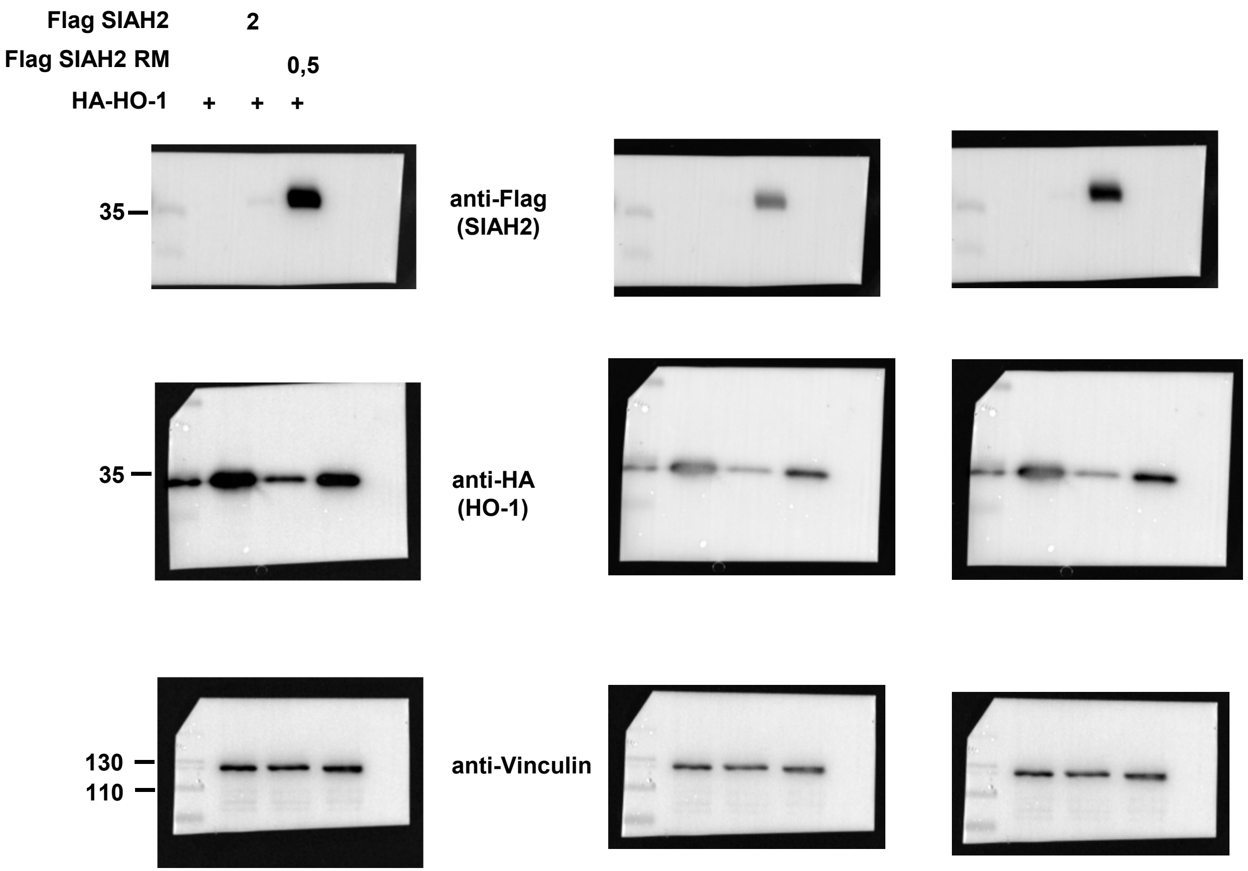

Flag SIAH2
2
Flag SIAH2 RM
0,5
HA-HO-1
+ + +
35
anti-Flag
(SIAH2)
35
anti-HA
(HO-1)
130
110
anti-Vinculin

Figure 5D

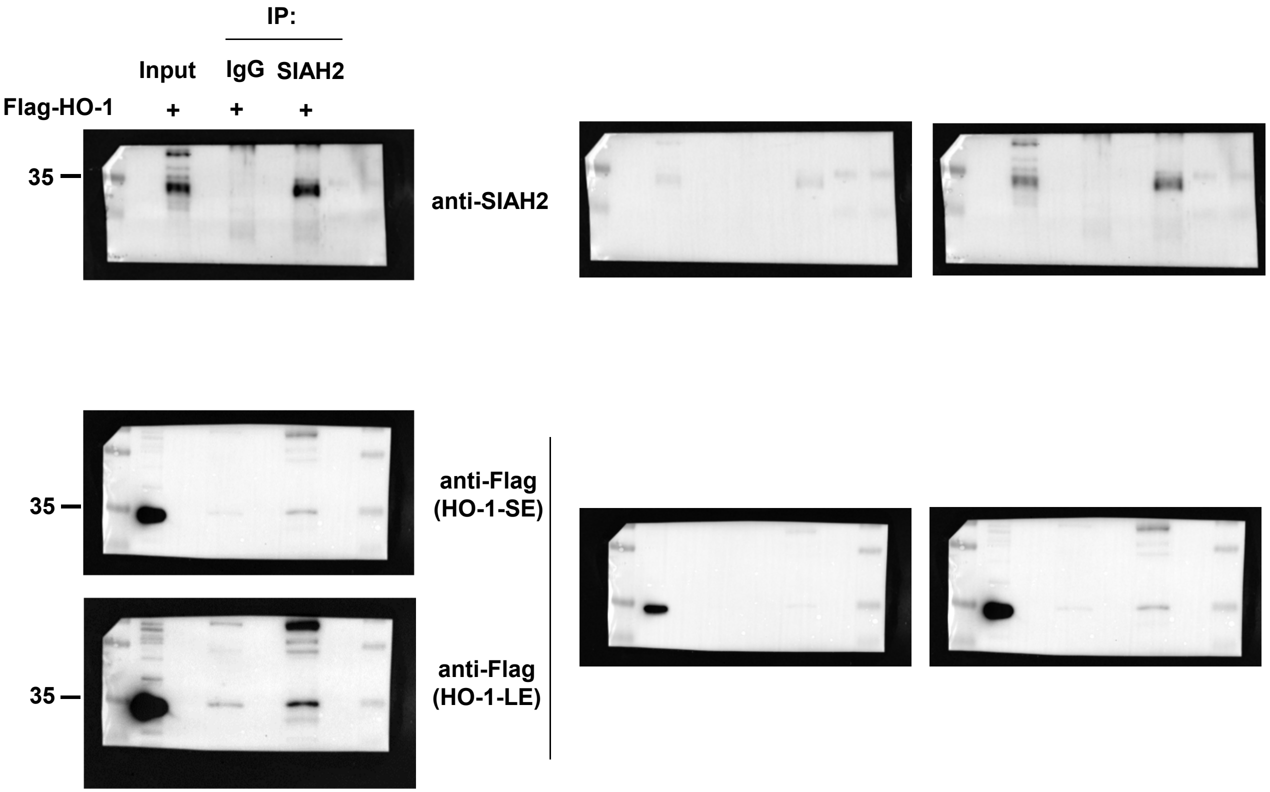

IP:
Input
IgG
SIAH2
Flag-HO-1
+
+
+
35
anti-SIAH2
35
anti-Flag
(HO-1-SE)
35
anti-Flag
(HO-1-LE)

Figure 5D

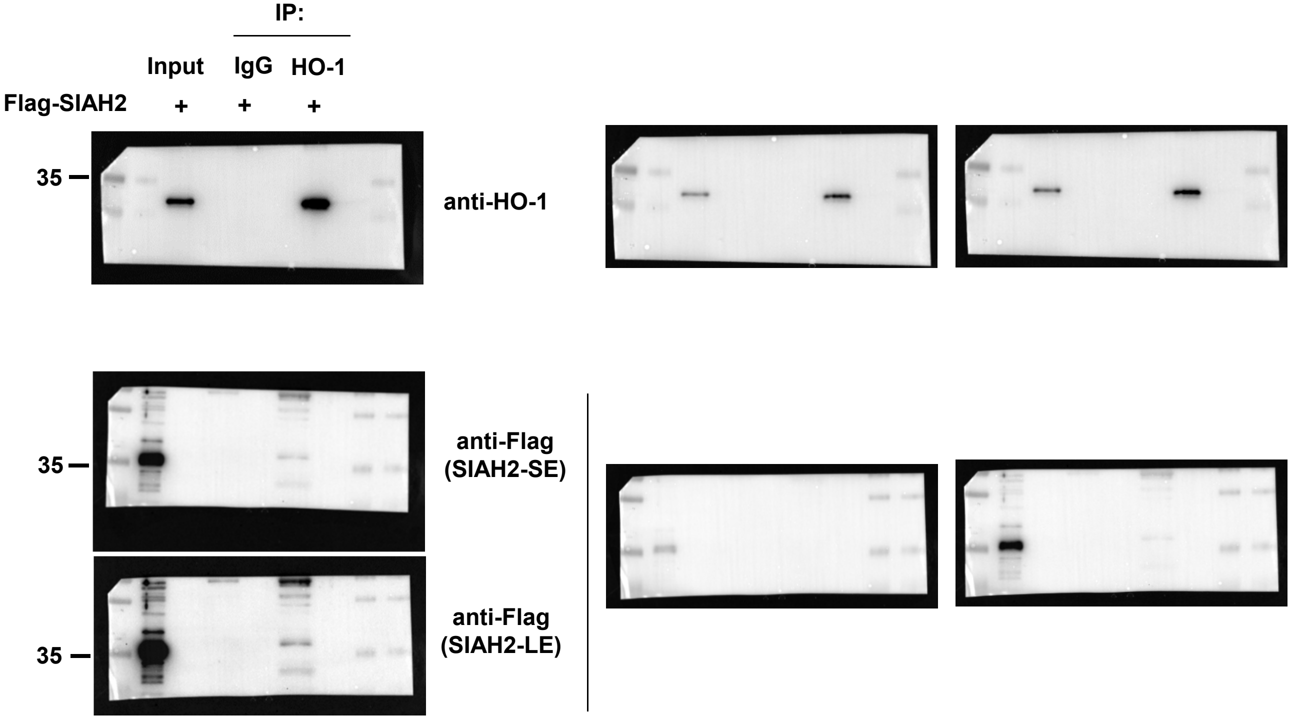
IP:
Input
IgG
HO-1
Flag-SIAH2
+
+
+
35
anti-HO-1
35
anti-Flag
(SIAH2-SE)
35
anti-Flag
(SIAH2-LE)

Figure 6A

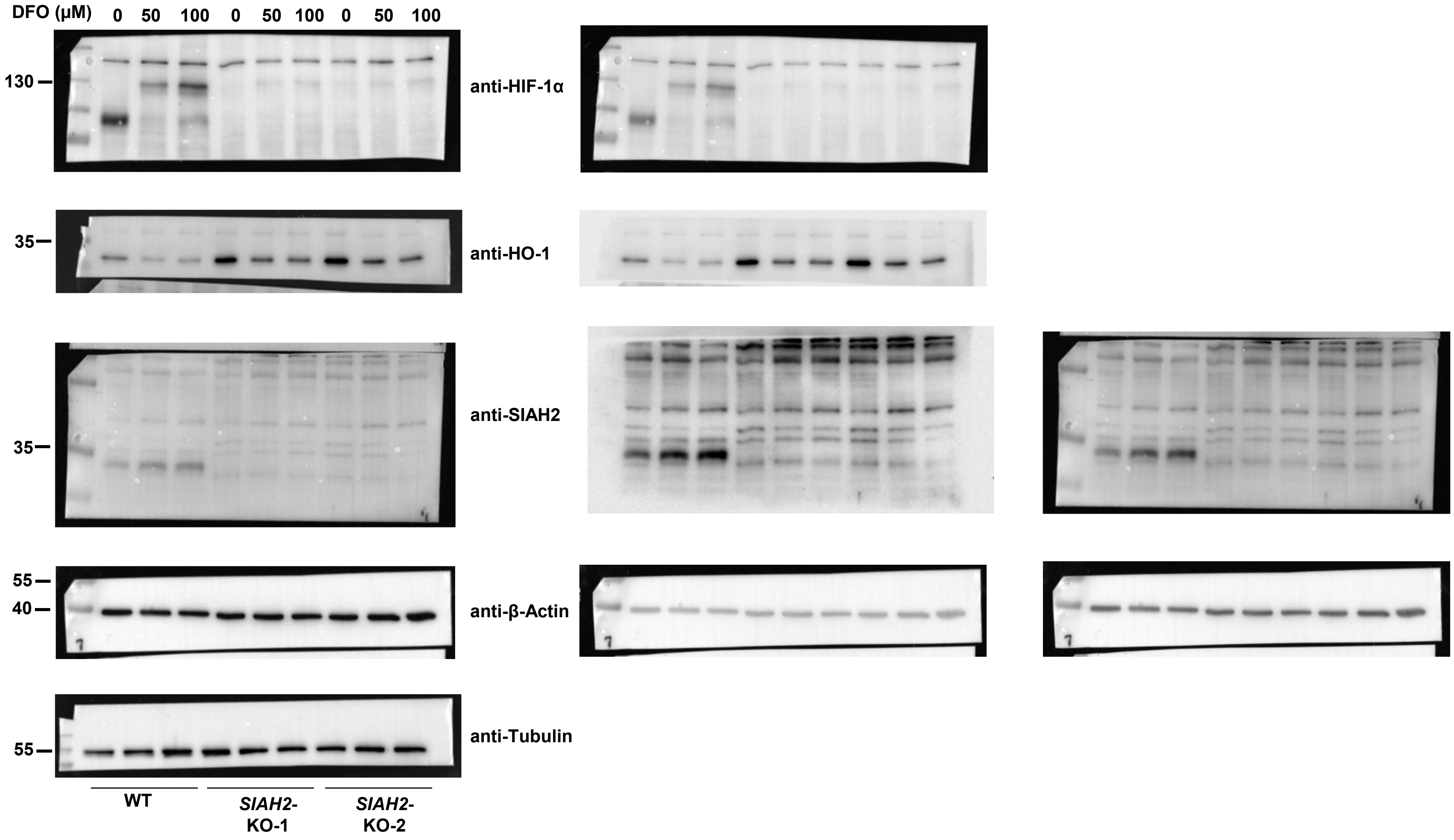
DFO (μM) 0 50 100 0 50 100 0 50 100
130
anti-HIF-1α
35
anti-HO-1
anti-SIAH2
35
55
40
anti-β-Actin
anti-Tubulin
55
WT
SIAH2-KO-1
SIAH2-KO-2

Figure 7A

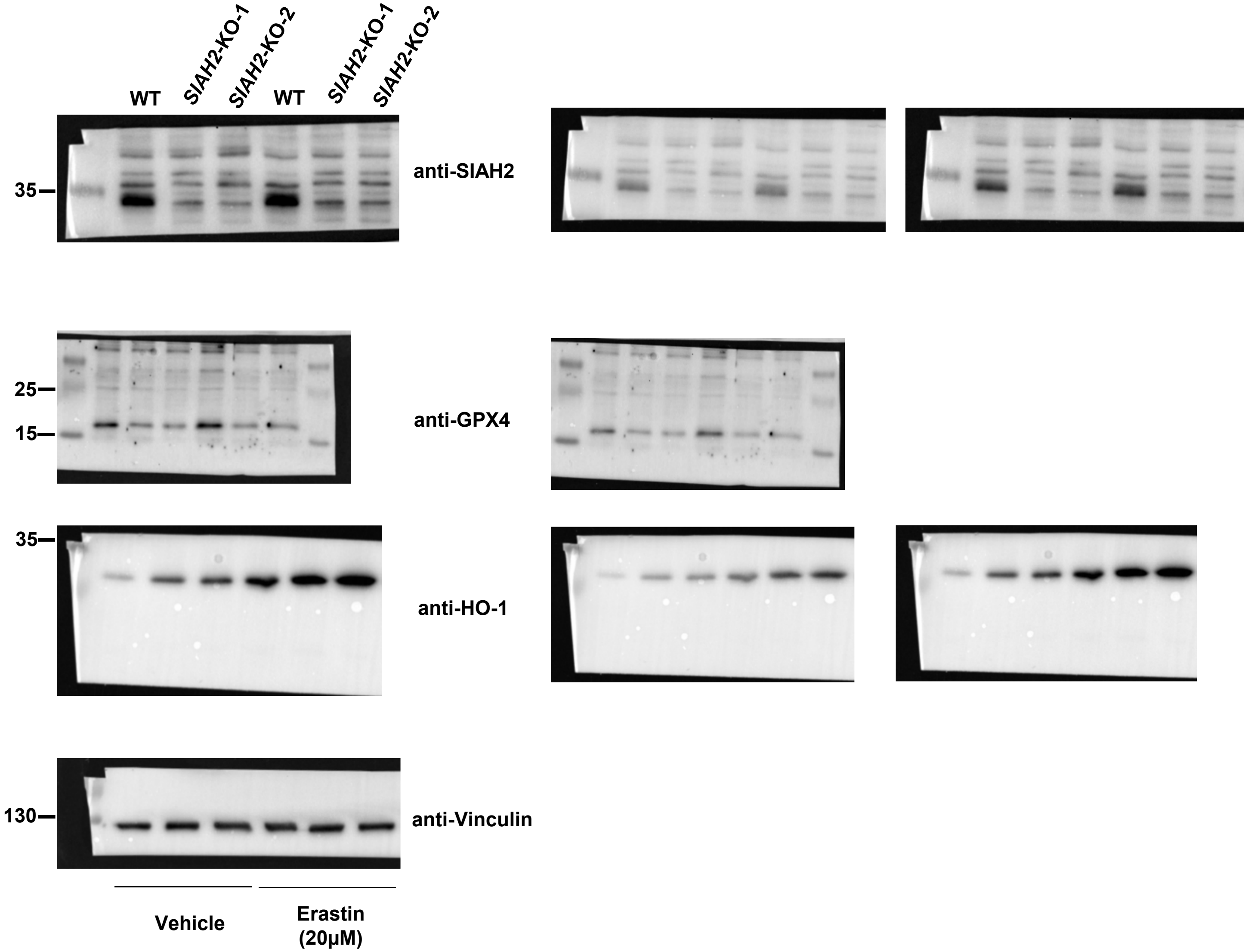
WT
SIAH2-KO-1
SIAH2-KO-2
WT
SIAH2-KO-1
SIAH2-KO-2
35
anti-SIAH2
25
15
anti-GPX4
35
anti-HO-1
130
anti-Vinculin
Vehicle
Erastin (20μM)

Figure 7B

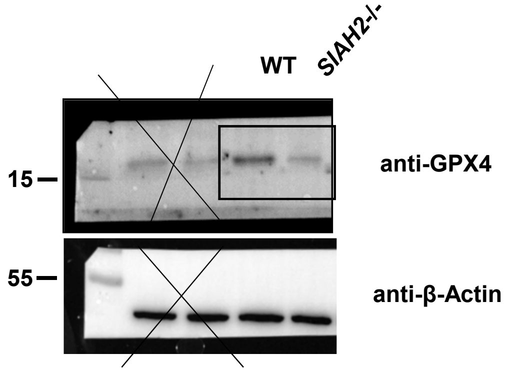

Figure 7C

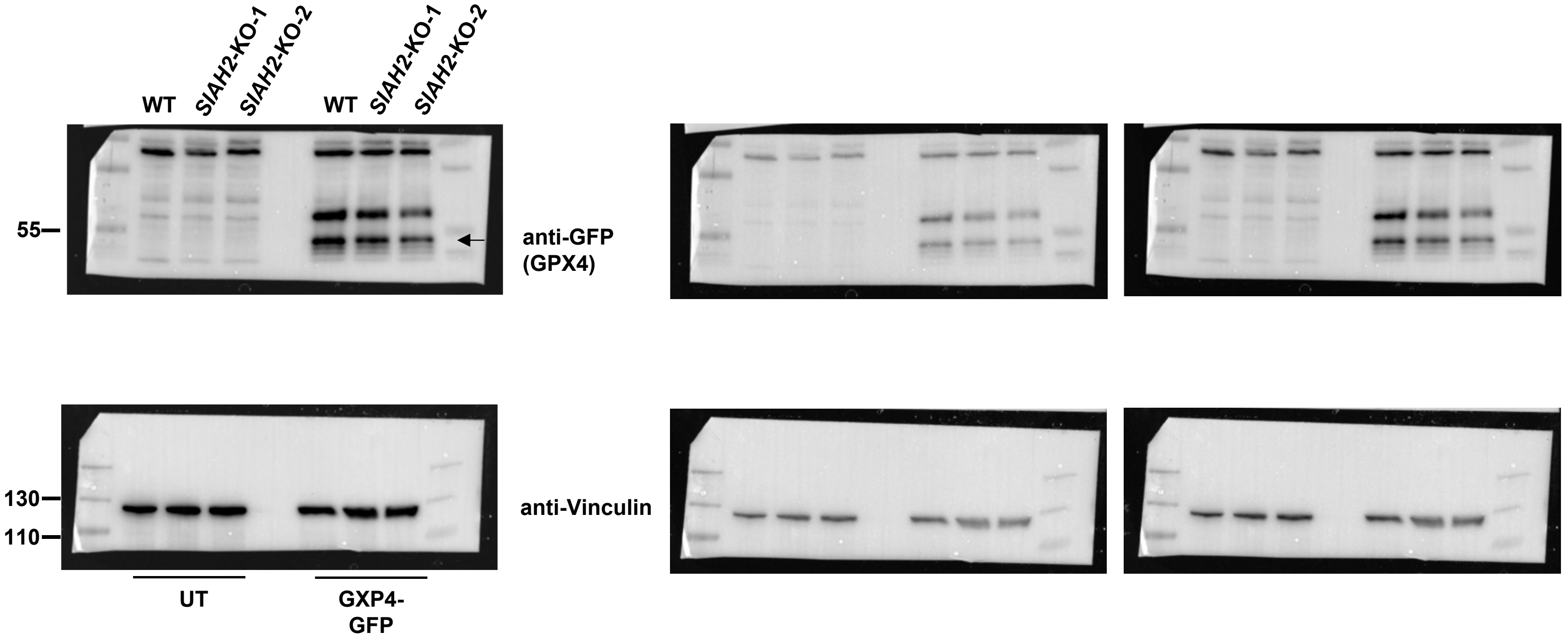

Figure S3

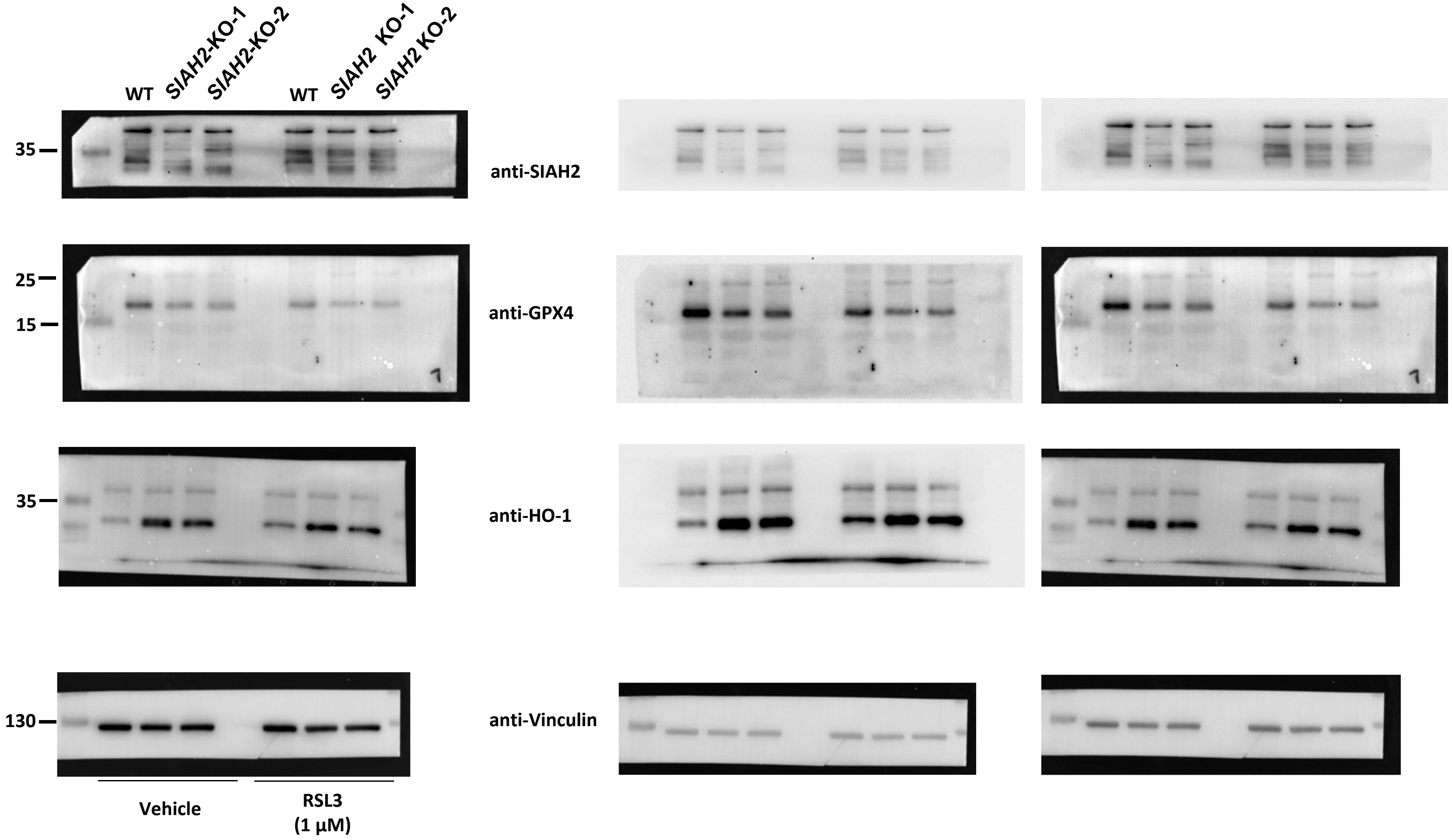
WT
SIAH2-KO-1
SIAH2-KO-2
WT
SIAH2 KO-1
SIAH2 KO-2
35
anti-SIAH2
25
15
anti-GPX4
35
anti-HO-1
130
anti-Vinculin
Vehicle
RSL3
(1 μM)
