# Supplementary material for: SIAH2-mediated and organ-specific restriction of HO-1 expression by a dual mechanism
Source: Sci Rep. 2020 Feb 10;10:2268. doi: 10.1038/s41598-020-59005-3 (PMC7010731; doi:10.1038/s41598-020-59005-3)
Supplement: Supplementary file 2 — Supplemental Information2. [file 41598_2020_59005_MOESM2_ESM.pdf]

Chillappagari et al. supplementary table 1

Antibodies

Primary antibody (clone)	Species	Supplier
HO-1	rabbit pAb	Protein tech #10701-1-AP
Siah2	rabbit pAb	Protein tech #12651-1-AP
GPX4	rabbit pAb	Protein tech #14432-1-AP
Flag (M2)	mouse mAb	Sigma Aldrich #F3165-1
HA (3F10)	rat pAb	Roche #11867423001
β-tubulin	mouse mAb	DSHB #E7
Vinculin	mouse mAb	Sigma # V9131
β-Actin	rabbit pAb	Abcam #ab8227
normal Rabbit IgG	rabbit	Cell Signalling #2729S
HIF-1α	rabbit pAb	Cell Signalling #14179
GAPDH	rabbit pAb	Cell Signaling #2118
CD31	rat mAb	BioRad #MCA2388
Turbo-GFP	rabbit pAb	Thermo #PA5-22688

Secondary antibody	Conjugated to	Supplier
goat-anti-rabbit IgG	alexa fluor-488-Green	Dianova #111-545-003
goat-anti-rabbit IgG	HRP	Dianova #111-035-144
goat-anti-mouse IgG	HRP	Dianova #112-035-143

Plasmids

Plasmid	Origin	Reference
pX459	F. Zhang	PMID: 24157548
pX459-sgRNA hu SIAH2	this study	
HA-Siah2 WT	M. Calzado Canale	PMID: 26751770
FLAG-Siah2	M.L. Schmitz	PMID: 23044042
FLAG-Siah2 RM	M.L. Schmitz	PMID: 23044042
HA-Siah1	M.L. Schmitz	PMID: 23044042
pINDUCER10	M.L. Schmitz	PMID: 28615693
pINDUCER10-Luci	M.L. Schmitz	PMID: 28615693

pINDUCER10-Siah2	this study	
mTert-pBabe-puro	J. Bidwell	PMID: 21732358
HA-HO-1	P. Lehner	PMID: 24958774
Flag-HO-1	L.Y. Chau	PMID: 24931165
GFP-GPX4	Ori-GENE	RG208065

DNA-Oligonucleotides for CRISPR/Cas9 and pIND-mediated SIAH2 knock-out and knock-down

Oligo name	Sequence (5´ to 3´)
px459-huSIAH2-for	CACCGCTGCAGGGTTTATTAGCGC
px459-SIAH2-rev	AAACGCGCTAATAAACCCTGCAGC
pIND-SIAH2-for	TCGAGAAGGTATATTGCTGTTGACAGTGAGCGCTAATGGGAACCTTGGAATCAATAGTGAAGCCACAGATGTATTGATTCCAAGGTTCCCATTATTGCCTACTGCCTCGGACTTCAAGGGGCTAG
pIND-SIAH2-rev	AATTCTAGCCCCTTGAAGTCCGAGGCAGTAGGCAATAATGGGAACCTTGGAATCAATACATCTGTGGCTTCACTATTGATTCCAAGGTTCCCATTATCGCTCACTGTCAACAGCAATATACCTTC

DNA-Oligonucleotides for qPCR

Oligo name	Sequence (5´ to 3´)
Hu-HO1-FP	TTCTTCACCTTCCCCAACATTG
Hu-HO1-RP	CAGCTCCTGCAACTCCTCAAA
Hu-β-actin-FP	CATGTACGTTGCTATCCAGGC
Hu-β-actin-RP	CTCCTTAATGTCACGCACGAT
Hu-GPX4-f1	CTTTGCCGCCTACTGAAGCC
Hu-GPX4-r1	TCCAGGTTAACCATGTGCCCG
Hu-GPX4-f2	TTCCCGTGTAACCAGTTCGG
Hu-GPX4-r2	GCCCTTGGGTTGGATCTTCA
Ms-HO-1-FP	GGTACACATCCAAGCCGAGAATGCT
Ms-HO-1-RP	GCAGCTCCTCAGGGAAGTAGAGT
Ms-β-actin-FP	CTCCCTGGAGAAGAGCTATGA
Ms-β-actin-RP	CAGGATTCCATACCCAAGAAGG
Primer for Siah2 genomic PCR	AGTGCATCAGCGCGGTCCA

Primer for Siah2 genomic PCR	AAGCAGACCGGACACTCGAAG

Reagents

**Name**	**working concentration**	**Supplier**
Anisomycin	5 µM	Sigma Aldrich #A9789
Lactacystin	0.5 and 2 µM	Santa Cruz #SC-3575
Puromycin	1 and 2 µg/ml	Invivogen #anti-pr-1
Desferroxamine	50 and 100 µM	Sigma Aldrich #D9533
Erastin	20 µM	Cayman Chemicals #17754
Doxycycline	1 µg/ml	Sigma Aldrich #D9891
Polybrene	5 µg/ml	Sigma Aldrich #H9268
Collagenase	400 mg/l	Biochrom AG #C 1-28
Polyethylenimine	3 µg/µg DNA	PolyScience #23966
RSL3	1 µM	Sigma Aldrich #SML2234
Ferrostatin	1 µM	Sigma Aldrich #SML0583
